# Regulation of *MCCC1* expression by a Parkinson’s disease-associated intronic variant: implications for pathogenesis

**DOI:** 10.1038/s10038-025-01335-z

**Published:** 2025-04-11

**Authors:** Shunsaku Sogabe, Hiroko Nakano, Yusuke Ogasahara, Pei-Chieng Cha, Yuko Ando, Mariko Taniguchi-Ikeda, Ryusaku Matsumoto, Motoi Kanagawa, Kazuhiro Kobayashi, Shigeo Murayama, Takashi Aoi, Tatsushi Toda, Wataru Satake

**Affiliations:** 1 Division of Neurology/Molecular Brain Science, Kobe University Graduate School of Medicine, Kobe, Hyogo 650-0017 Japan; 2 Department of Genomic Medicine, Research Institute, National Cerebral and Cardiovascular Center, Suita, Osaka 564-8565 Japan; 3 Department of Cell Biology and Molecular Medicine, Ehime University Graduate School of Medicine, Toon, Ehime 791-0295 Japan; 4 Department of Clinical Genetics, Fujita Health University Hospital, Toyoake, Aichi 470-1192 Japan; 5 Division of Stem Cell Medicine, Kobe University Graduate School of Medicine, Kobe, Hyogo 650-0017 Japan; 6 Department of Life Science Frontiers, Center for iPS Cell Research and Application, Kyoto University, Kyoto, Kyoto, 606-8507 Japan; 7 Department of Neuropathology (Brain Bank for Aging Research), Tokyo Metropolitan Institute for Geriatrics and Gerontology, Itabashi-ku, Tokyo 173-0015 Japan; 8 Brain Bank for Neurodevelopmental, Neurological, and Psychiatric Disorders, Molecular Research Center for Children’s Mental Development, United Graduate School of Child Development, Osaka University, Suita, Osaka 565-0871 Japan; 9 Department of Neurology, Graduate School of Medicine, The University of Tokyo, Bunkyo-ku, Tokyo 113-8655 Japan

**Keywords:** Parkinson's disease, Genome-wide association studies

## Abstract

Parkinson’s disease (PD) is a common neurodegenerative disorder characterized by dopaminergic neuron loss and α-synuclein aggregation. While some familial cases result from single-gene mutations, most are sporadic, involving complex genetic and environmental interactions. Among PD risk loci identified through genome-wide association studies, *MCCC1* encodes a mitochondrial enzyme essential for leucine catabolism; however, the causal variant remains unclear. Here, we investigated whether the intronic variant rs12637471 regulates *MCCC1* mRNA expression and influences PD risk. Postmortem brain analysis revealed significantly elevated *MCCC1* mRNA levels in G-allele carriers, consistent with peripheral tissue eQTL data from GTEx. Using CRISPR/Cas9-edited induced pluripotent stem cells, we generated isogenic lines differing only at rs12637471 and observed increased *MCCC1* expression in G-allele dopaminergic neurons. Given MCCC1’s mitochondrial role, its dysregulation may impact mitochondrial homeostasis, autophagy, or inflammation, potentially contributing to PD pathogenesis.

Parkinson’s disease (PD) is the second most prevalent neurodegenerative disorder, affecting millions worldwide. Clinically, PD manifests as bradykinesia, resting tremor, and rigidity. Pathologically, it is characterized by dopaminergic neuron loss in the substantia nigra pars compacta and Lewy body accumulation, primarily composed of α-synuclein. While a subset of familial PD cases results from monogenic mutations, most cases are sporadic, arising from complex genetic and environmental interactions. Genome-wide association studies (GWAS) have identified multiple PD risk loci across diverse populations [1–3]. However, pinpointing causal variants and elucidating their biological effects remain key challenges [4–6].

Among these loci, *MCCC1* risk alleles have been associated with PD across populations, highlighting the gene’s robust link to disease susceptibility [1, 3]. *MCCC1* encodes the α-subunit of 3-methylcrotonyl-CoA carboxylase, a mitochondrial enzyme essential for leucine catabolism [7, 8]. Despite this association, the functional variant underlying PD risk and its molecular mechanism remain unclear. Here, we explored whether the allelic difference of rs12637471, an intronic *MCCC1* variant, regulates *MCCC1* mRNA expression and contributes to PD risk using public datasets, human postmortem brain tissue, and induced pluripotent stem cell (iPSC)-derived dopaminergic neurons.

In European GWAS, rs12637471 G-A, an intronic MCCC1 variant, showed the strongest association with PD (*p* = 2.14 × 10^−21^) and A allele of rs12637471 showed a protective effect against PD (odds ratio = 0.842) [1]. Similarly, in Asian cohorts, rs2292056—a SNP in strong linkage disequilibrium with rs12637471—was identified as the most significant (*p* = 8.14 × 10^−17^) [3]. Public datasets indicate that rs12637471 resides in an evolutionary conserved sequence in mammals, overlapping histone modifications typical of active enhancer H3K27ac (Supplementary Fig. 1A, B). Furthermore, expression quantitative trait locus (eQTL) data from the GTEx project reveal that the G allele correlates with elevated *MCCC1* mRNA expression in peripheral tissues (Supplementary Fig. 1C), suggesting a role as a cis-regulatory enhancer. Based on these findings, we hypothesize that the G allele of rs12637471 directly influences *MCCC1* expression and, consequently, contributes to PD risk.

To determine whether rs12637471 affects *MCCC1* expression in the central nervous system, we analyzed postmortem frontal cortex samples from 31 individuals without neuropathological abnormalities from the Brain Bank for Aging Research. Genotyping revealed 4 GG, 12 AG, and 15 AA individuals at rs12637471. We extracted total RNA and performed quantitative reverse-transcription PCR (qRT-PCR) using TaqMan assays specific to *MCCC1*. Carriers of the G allele (AG and GG) exhibited significantly elevated *MCCC1* mRNA expression—1.14-fold and 1.20-fold higher than AA homozygotes, respectively (Fig. 1A). This observation aligns with GTEx eQTL data from peripheral tissues (Supplementary Fig. 1C), reinforcing the role of rs12637471 in regulating *MCCC1* transcription.

**Fig. 1:**
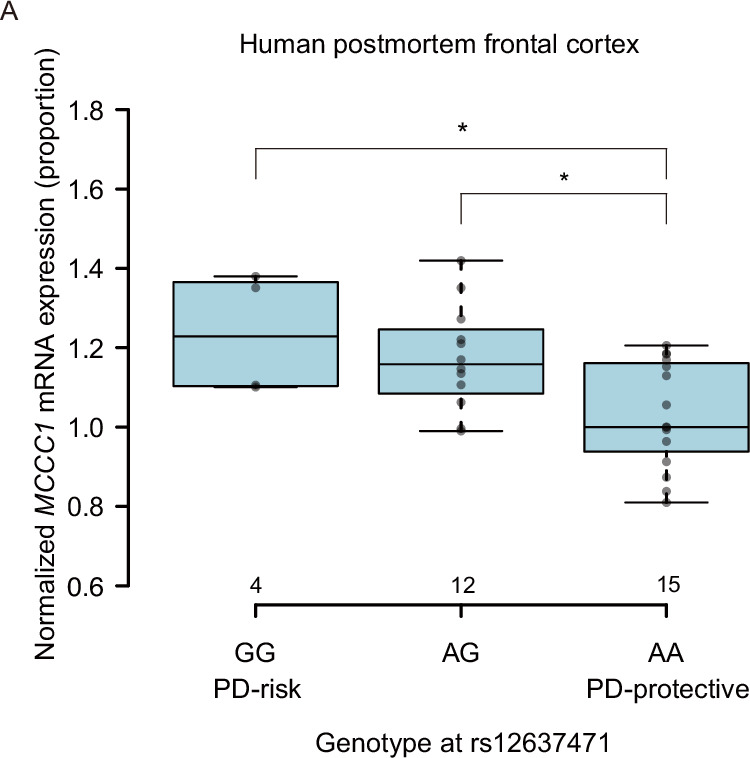
Risk allele G–associated upregulation of *MCCC1* mRNA in human postmortem brain and iPSC-derived isogenic neurons. **A** qRT-PCR analysis of *MCCC1* mRNA expression in postmortem frontal cortex, stratified by rs12637471 genotype. Center lines show the medians and box limits indicate the 25th and 75th percentiles. Whiskers extend 1.5 times the interquartile range from the 25th and 75th percentiles, outliers are represented by dots. Statistical significance was determined using one-way ANOVA followed by Tukey’s multiple comparison test. *P < 0.05 vs. the protective-allele **A** homozygote group (GG: *n* = 4, AG: *n* = 12, AA: *n* = 15)

To establish a direct causal link, we used CRISPR/Cas9 genome editing in human iPSCs derived from the 201B7 cell line [9–12]. The genotype of rs12637471 was AG in the wild-type iPSC 201B7. Guide RNAs targeted rs12637471, and a synthetic single-stranded oligodeoxynucleotide template facilitated homology-directed repair. Clonal expansion and genotyping confirmed AA, AG, and GG clones via TaqMan assays and Sanger sequencing (Fig. 2A). Among 997 colonies, 18 clones yielded the PD-protective (AA) genotype, while 23 clones among 551 colonies exhibited the PD-risk (GG) genotype (Fig. 2B). These isogenic lines, differing only at rs12637471, provided an ideal model to assess its direct impact on *MCCC1* expression (Fig. 2C).

**Fig. 2:**
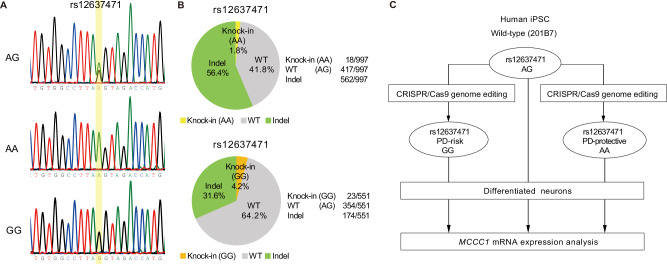
Generation of isogenic iPSC lines differing only at rs12637471 using CRISPR/Cas9-mediated genome editing. **A** Sanger sequencing chromatograms of genome-edited iPSC lines targeting rs12637471. “AG” represents the wild-type (201B7) genotype, while “AA” and “GG” indicate single-nucleotide knock-in clones. The rs12637471 position is highlighted in yellow. **B** Pie charts depicting the knock-in outcomes (AA or GG) from CRISPR/Cas9-mediated editing of wild-type AG iPS cells at rs12637471, as determined by TaqMan genotyping and Sanger sequencing. **C** Schematic representation of isogenic cell-line generation from single human iPS cells using CRISPR/Cas9-based genome editing, followed by neuronal differentiation and *MCCC1* mRNA expression analysis

We differentiated these isogenic iPSC lines into dopaminergic neurons using a dual SMAD inhibition protocol promoting floor plate induction (Fig. 3A) [10–12]. Neuronal differentiation efficiency was assessed by quantifying tyrosine hydroxylase (TH) + /FOXA2+ and TH + /TUJ1+ double-positive dopaminergic neurons, revealing no significant genotype-dependent differences in TH + /FOXA2+ (12.6 ± 2.8% [AA], 13.9 ± 0.7% [AG], 12.4 ± 2.7% [GG]) or TH + /TUJ1+ (14.6 ± 2.0% [AA], 15.5 ± 3.6% [AG], 15.1 ± 2.4% [GG]) cells at day 42 (mean ± SE, *n* = 3) (Fig. 3B, C). These findings confirm uniform differentiation across genotypes. qRT-PCR analysis of *MCCC1* mRNA expression in differentiated neurons revealed significantly elevated levels in G-allele carriers, with 1.25-fold and 1.32-fold increases in AG and GG genotypes, respectively, compared to AA (Fig. 3D). These results, consistent with postmortem brain data, confirm that rs12637471 enhances *MCCC1* transcription in dopaminergic neurons.

**Fig. 3:**
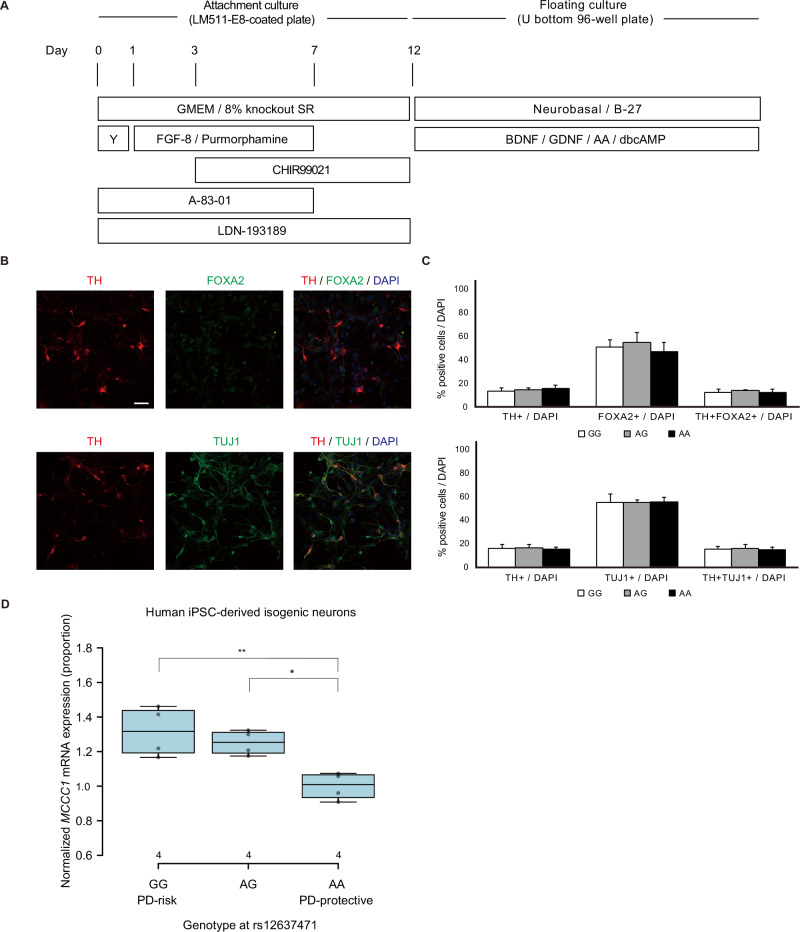
Analysis of in vitro–differentiated human iPSC-derived neurons. **A** Overview of the neural differentiation protocol from human iPS cells. **B** Immunostaining of differentiated neurons on day 42, showing TH/FOXA2/DAPI (top) and TH/TUJ1/DAPI (bottom). Insets highlight double-positive cells. Scale bar, 50 μm. **C** Quantification of TH + /FOXA2+ and TH + /TUJ1+ cells as a percentage of total cells on day 42. Data are presented as mean ± SE (AA: *n* = 3, AG: *n* = 3, GG: *n* = 3). **D** qRT-PCR analysis of *MCCC1* mRNA expression in iPSC-derived isogenic neural cultures on day 42, stratified by rs12637471 genotype. Center lines show the medians and box limits indicate the 25th and 75th percentiles. Whiskers extend 1.5 times the interquartile range from the 25th and 75th percentiles, outliers are represented by dots. Statistical significance was determined using one-way ANOVA followed by Tukey’s multiple comparison test. *P < 0.05; **P < 0.01 vs. the protective-allele **A** homozygote group (GG: *n* = 4, AG: *n* = 4, AA: *n* = 4)

Although the precise downstream effects of MCCC1 upregulation in PD remain unexplored, several mechanisms are plausible. First, MCCC1 localizes to mitochondria, forms a complex with MCCC2, and participates in leucine catabolism, suggesting that its dysregulation may impair mitochondrial function—an established contributor to PD. Second, leucine-derived metabolites, such as acetyl-CoA, regulate autophagy [8], and altered autophagic flux may disrupt α-synuclein homeostasis. Third, aberrant MCCC1 activity may trigger chronic inflammation under specific conditions [13]. Further investigation is required to elucidate these potential pathways.

Our findings align with prior studies demonstrating that functional intronic variants can profoundly influence gene expression and disease risk [5, 6]. Notably, Soldner et al. identified intronic regulatory variants in *SNCA* affecting α-synuclein expression [5], while Gupta et al. reported a cis-regulatory variant in *PHACTR1* associated with vascular disease [6]. These findings underscore the importance of transcriptional regulation in disease susceptibility, beyond coding variants. The consistent association of *MCCC1* with PD across populations further emphasizes the need to characterize regulatory polymorphisms such as rs12637471.

Our study identifies rs12637471 as a functional variant within the *MCCC1* locus that enhances *MCCC1* transcription in human brain tissue and iPSC-derived dopaminergic neurons. This upregulation may disrupt mitochondrial and metabolic homeostasis, and contribute to PD risk. Future studies should assess whether manipulating *MCCC1* expression—via knockdown, pharmacological inhibition, or other strategies—can mitigate PD-like phenotypes, particularly in the context of α-synuclein aggregation and mitochondrial dysfunction. Understanding the interplay between MCCC1, leucine metabolism, and dopaminergic neuron survival will be essential for developing novel therapeutic approaches for PD. More broadly, our findings highlight the value of integrating GWAS signals, molecular biology tools, and patient-derived models to decipher the genetic architecture of sporadic neurodegenerative diseases.

## Materials and methods

Full details of the experimental materials and methods—including tissue processing, genome-editing protocols, and neuronal differentiation procedures—are provided in the Supplementary Information.

## Supplementary information

Supplementary Information

Supplementary Figure 1

## Data Availability

The data supporting the findings of this study are available upon request from the corresponding author.
