# Supplementary material for: Regulation of MCCC1 expression by a Parkinson’s disease-associated intronic variant: implications for pathogenesis
Source: J Hum Genet. 2025 Apr 11;70(7):371–4. doi: 10.1038/s10038-025-01335-z (PMC12137145; doi:10.1038/s10038-025-01335-z)
Supplement: Supplementary file 1 — Supplementary Information [file 10038_2025_1335_MOESM1_ESM.docx]

**Materials and Methods**

**Human Postmortem Brain Samples**

Thirty-one frontal cortex samples (mean age, 77.8 years; no significant neuropathological findings) were provided by the Brain Bank for Aging Research (https://www.tmghig.jp/research/en/eresearch/researchteam/vice01/team-05/a23/). Genomic DNA from brain samples were genotyped for rs12637471 (AA, AG, GG) using TaqMan assays (Applied Biosystems), with genotypes confirmed by Sanger sequencing.

**CRISPR/Cas9 Genome Editing in Human iPSCs**

The human iPSC line 201B7 (RIKEN BRC) was maintained under feeder-free conditions in StemFit medium and passaged using TrypLE Select. Guide RNAs targeting rs12637471 were cloned into the PX459 vector (Addgene). A single-stranded oligodeoxynucleotide (ssODN) template in Lipofectamine Stem Reagent (Invitrogen) was used to facilitate homology-directed repair. Following puromycin selection and clonal expansion, genotypes were verified by TaqMan assays and Sanger sequencing. Isogenic iPSC lines carrying AA, AG, or GG genotypes at rs12637471 were selected for further analysis. Off-target effects were not assessed experimentally. Sequence information for the guide RNAs and ssODN we used in this study is as follows.

Guide RNA sequences for rs12637471 G>A: AAAGCCTCATGGTCTAC**T**TAAGG (PAM)

Guide RNA sequences for rs12637471 A>G: AAAGCCTCATGGTCTAC**C**TAAGG (PAM)

ssODN sequences for rs12637471 G>A:

GATCTGAAACCGAATGATGGCCTGGCCCAGAATGCTGTGGCCTTA**A**GTAGACCATGAGGCTTTGACCTTGAGGTGATGTGGTTGTGAGCA (90bp)

ssODN sequences for rs12637471 A>G:

GATCTGAAACCGAATGATGGCCTGGCCCAGAATGCTGTGGCCTTA**G**GTAGACCATGAGGCTTTGACCTTGAGGTGATGTGGTTGTGAGCA (90bp)

**Neuronal Differentiation**

Isogenic iPSCs were differentiated into dopaminergic neurons using a modified dual SMAD inhibition approach. Small-molecule inhibitors (LDN193189, A83-01) and growth factors (FGF8, purmorphamine) were applied to induce floor plate precursors. Cells were subsequently transferred to Neurobasal medium supplemented with B27, GDNF, BDNF, and dbcAMP and maintained for approximately six weeks before analysis [1–3].

**Gene Expression Analysis**

Total RNA was extracted using the RNeasy Mini Kit (Qiagen), and cDNA synthesis was performed with SuperScript IV (Invitrogen). Quantitative PCR was conducted using TaqMan gene expression assays for *MCCC1*, with *GAPDH* or *ACTB* serving as internal controls. Relative expression was assessed using the ΔΔCt method, and statistical significance was determined by ANOVA followed by post-hoc tests [4,5].

**Immunofluorescence staining**

Differentiated neurons were fixed with 4% paraformaldehyde in PBS and blocked with 0.2% Triton X-100 and 5% fetal bovine serum in PBS. Nuclei were visualized using VECTASHIELD Vibrance Antifade Mounting Medium with DAPI (VECTOR Laboratories). The following primary antibodies were used: anti-FOXA2 (R&D, AF2400, 1:500), anti-TUJ1 (Covance, MMS-435P, 1:600), and anti-TH (Millipore, AB152, 1:400). Images were acquired using a fluorescence microscope Keyence BZ-9000 at 20 × magnification, and positively stained cells were quantified using ImageJ.

**Statistical Analysis**

Data are presented as mean ± standard error. One-way ANOVA followed by Tukey’s multiple comparison test was used for genotype comparisons unless otherwise specified.

**Ethics Approval**

This study was reviewed and approved by the Ethics Committee of Kobe University Graduate School of Medicine and the Faculty of Medicine of the University of Tokyo. The contribution of the Brain Bank for Aging Research was approved by the Institutional Review Board (IRB) of the Tokyo Metropolitan Institute for Geriatrics and Gerontology.

**Supplementary References**

1. Doi D, Samata B, Katsuki T, et al. Isolation of human induced pluripotent stem cell-derived dopaminergic progenitors by cell sorting for successful transplantation. Stem Cell Reports. 2014;2:337–50.
2. Kikuchi T, Morizane A, Doi D, et al. Human iPS cell-derived dopaminergic neurons function in a primate Parkinson’s disease model. Nature. 2017;548:592–6.
3. Ishikawa T, Imamura K, Egawa N, et al. Genetic and pharmacological correction of aberrant dopamine synthesis using patient iPSCs with BH4 metabolism disorders. Hum Mol Genet. 2016;25:5188–99.
4. Mizuta I, Satake W, Nakabayashi Y, et al. Multiple candidate gene analysis identifies alpha-synuclein as a susceptibility gene for sporadic Parkinson’s disease. Hum Mol Genet. 2006;15:1151–8.
5. Soldner F, Stelzer Y, Shivalila CS, et al. Parkinson-associated risk variant in distal enhancer of α-synuclein modulates target gene expression. Nature. 2016;533:95–9.
