# Supplementary material for: Regulation of MCCC1 expression by a Parkinson’s disease-associated intronic variant: implications for pathogenesis
Source: J Hum Genet. 2025 Apr 11;70(7):371–4. doi: 10.1038/s10038-025-01335-z (PMC12137145; doi:10.1038/s10038-025-01335-z)
Supplement: Supplementary file 2 — Supplementary Figure 1 [file 10038_2025_1335_MOESM2_ESM.docx]

**Supplementary Figure 1. Genomic characteristics of *MCCC1* from publicly available databases** (A) Evolutionary conservation of the rs12637471 locus. (B) Chromatin immunoprecipitation sequencing (ChIP-seq) for H3K27ac signal at the *MCCC1* locus, indicating a potential promoter/enhancer region and a transcription factor–binding hotspot at rs12637471. (C) Expression quantitative trait locus (eQTL) analysis of *MCCC1* expression in human tibial nerve and skeletal muscle from the GTEx project (<https://gtexportal.org/>), stratified by rs12637471 genotype.
